# Left Atrial Appendage Occlusion in High Bleeding Risk Patients

**DOI:** 10.1155/2019/6704031

**Published:** 2019-02-18

**Authors:** Pierluigi Merella, Giovanni Lorenzoni, Alessandro P. Delitala, Filomena Sechi, Federica Decandia, Graziana Viola, Paola Berne, Gianluca Deiana, Patrizio Mazzone, Gavino Casu

**Affiliations:** ^1^Department of Cardiology, Ospedale San Francesco, Nuoro, Italy; ^2^Istituto di Clinica Medica, Azienda Ospedaliero-Universitaria di Sassari, Sassari, Italy; ^3^Neurology Department and Stroke Unit, Ospedale San Francesco, Nuoro, Italy; ^4^Arrhythmology and Cardiac Pacing Unit, Cardiothoracic and Vascular Department, IRCCS Ospedale San Raffaele, Milan, Italy

## Abstract

**Objectives:**

The aim of this study was to investigate the outcomes of left atrial appendage occlusion (LAAO) in high bleeding risk patients suffering atrial fibrillation (AF) and to analyze the different antithrombotic therapies following the intervention.

**Background. Methods:**

This monocentric study included 68 patients with nonvalvular AF with an absolute contraindication to OAT or at high bleeding risk. Follow-up was done with a clinical visit at 3-6-12 months.

**Results:**

Successful LAAO was achieved in 67/68 patients. At discharge, 32/68 patients were on dual antiplatelet therapy (APT), 34/68 were without any antithrombotic therapy or with a single antiplatelet drug, and 2/68 were on anticoagulant therapy. At three-month follow-up visit, 73.6% of the patients did not receive dual APT, of whom 14.7% had no thrombotic therapy and 58.9% were on single antiplatelet therapy. During a follow-up of 1.4 ± 0.9 years, 3/62 patients had late adverse effects (2 device-related thrombus without clinical consequences and 1 extracranial bleeding). The device-related thrombosis was not related to the antithrombotic therapy.

**Conclusions:**

LAAO is feasible and safe and prevents stroke in patients with AF with contraindication to oral anticoagulant therapy. After LAAO, single antiplatelet therapy seems to be a safe alternative to dual antiplatelet therapy, especially in patients at high bleeding risk. No benefit has been observed with dual APT.

## 1. Introduction

Atrial fibrillation (AF) is the most common cardiac arrhythmia and its incidence and prevalence are constantly increasing [1]. The prevalence of AF in the general population is about 2% and increases with age, with a lifetime risk of about 15% [2]. Its prevalence has been projected to increase in the US to 12.1 million cases in 2030 [3].

AF is an independent risk factor for ischemic stroke and thromboembolic events, which significantly increase mortality and morbidity and can cause serious disabilities. Annual stroke rate in AF patients is about 5%, and at least 20% of all ischemic strokes are associated with AF [4]. Despite some risk factors have been identified [5–8], the natural history of its development is largely unpredictable. Oral anticoagulant therapy (OAT) is the cornerstone of management of AF patients at increased stroke risk [4]. CHA_2_DS_2_-VASc [9] is the thromboembolic risk assessment score recommended by the European Society of Cardiology [4], the American Heart Association [10], and the American College of Cardiology [10]. OAT is recommended in male patients with CHA_2_DS_2_-VASc ≥2 and in female patients with CHA_2_DS_2_-VASc ≥3; OAT should be considered in male patients with CHA_2_DS_2_-VASc =1 and in female patients with CHA_2_DS_2_-VASc =2. Two classes of antithrombotic drugs, Vitamin K Antagonists (VKA) and Nonvitamin K antagonist Oral Anticoagulants (NOACs), are recommended for the prevention of ischemic stroke in AF.

NOACs have proved equally effective with a lower risk of cerebral haemorrhage [11–15].

The decision to prescribe antithrombotic drugs must necessarily involve an assessment of the risk of stroke against the risk of major bleeding. The HAS-BLED [16] is the most widely used haemorrhagic risk score. A HAS-BLED score ≥3 identifies patients with high risk of haemorrhage, but this is not a criterion for exclusion from OAT [4]. Many other conditions confer an increased risk per se without affecting bleeding scores [17–22].

Left atrial appendage (LAA) occlusion offers an alternative mechanical approach [23, 24] to reduce cardioembolic risk in AF patients [25]. The rationale for LAA occlusion is based on the strong evidence that more than 90% of thrombi during nonvalvular AF originate in the LAA [26].

Currently, European guidelines recommended LAA occlusion in patients with AF and contraindications for long term anticoagulation (class IIb indication, level of evidence B) [4]. Clinical data are derived from real life registries due to the obvious difficulties to randomize patients with a contraindication to anticoagulation.

Compared to OAC therapy, LAA closure reduced the risk of life-threatening bleeding events, such as haemorrhagic stroke [27]. In the recently published EWOLUTION trial, LAA closure appeared safe and effective, obtaining an ischemic stroke rate as low as 1.1% [28]. Similarly, LAA closure with the ACP, now replaced by Amulet device, showed a favourable outcome for the prevention of AF-related thromboembolism [29].

After LAA occlusion, one of the most important problem is thrombus formation on the device surface.

Current manufacturer's instructions recommend the continuation of dual antiplatelet therapy (APT) for at least 3 months after the procedure. However, this therapeutic strategy might be a problem, considering their intrinsic high risk of bleeding.

In this study, we aimed to assess the feasibility and the safety of LAA closure and to compare the different strategies for postimplant antithrombotic therapy in a high bleeding risk population.

## 2. Methods

Sixty-eight patients underwent LAA occlusion between February 2014 and October 2017. All the procedures were performed at the regional referral Center of Ospedale San Francesco, Nuoro, Italy, by two operators (GC and PM). Eight patients were treated with ACP™ and AMPLATZER™ Amulet™ device (St. Jude Medical, St. Paul, MN, USA) and 60 patients with WATCHMAN™ device (Boston Scientific, Marlborough, MA, USA). Anthropometric and clinical data and indication to LAA closure were collected. CHA_2_DS_2_-VASc and HAS-BLED scores were calculated in each patient. Transesophageal echocardiography (TEE) was performed the day before the procedure to rule out LAA thrombus and to get accurate device sizing. Each procedure was performed under general anesthesia by femoral vein approach and transseptal puncture under fluoroscopic and TEE guidance. Five thousand units of heparin were administered intravenously. The activated clotting time was kept above 250 seconds during the procedure. Transthoracic echocardiography or TEE was performed at day 1 after implantation to confirm appropriate device implantation and to exclude residual device-related leak or thrombosis.

Technical success was defined as the successful implantation of device. Procedural success was defined as technical success without major procedure-related complications.

Postimplant antithrombotic was precribed and tailored according to the risk of bleeding of each patient. In particular, the choice of therapy was based on the history of previous major bleeding and global bleeding risk.

Events were labelled as “early” if they occurred within 7 days of the procedure or before discharge and “late” when they occurred after 7 days. Peripheral embolism, death, haemorrhagic stroke, and persistent need for OAT were also recorded.

Primary indication for LAA closure was considered in patients with two or more indications.

Patients signed a written informed consent before undergoing the procedure.

Patients were followed clinically at 1, 3, 6, and 12 months. TEE was performed at 3 and 12 months.

### 2.1. Statistical Analysis

Continuous traits were reported as mean and standard deviation. Categorical traits were reported as absolute frequency and percentages. The expected incidence of thromboembolic or bleeding events were calculated as the mean of each individual annual risk according to the patient's CHA_2_DS_2_-VASc and HAS-BLED scores. Thromboembolism reduction was calculated as follows: (expected %  −  observed % event rate)/expected % event rate. All analyses were conducted using Stata 12.0 statistical package.

## 3. Results

### 3.1. Patients

Mean age was 73.6 ± 8.7 years and 19.1% were female. The sample had a very high annual stroke risk (4.4%) and a very high estimated bleeding risk (5.8%), as shown in Table 1. Major comorbidities were ischemic heart disease (33.8%) and end-stage chronic kidney disease (33.8%).

**Figure 1:**
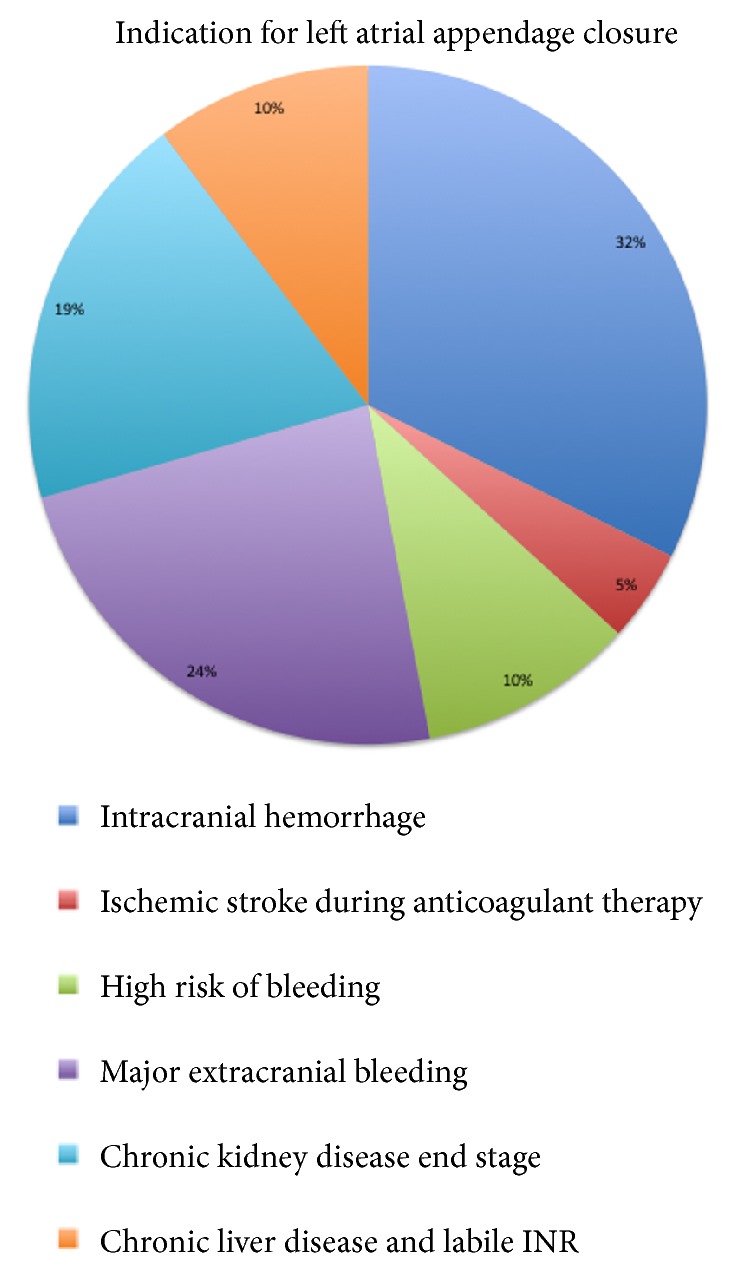
*Indication for left atrial appendage closure*. Main indications for LAA occlusion were a history of intracranial haemorrhage, high risk of bleeding, end-stage chronic kidney disease and chronic liver disease. All the patients had a contraindication to oral anticoagulant therapy.

Indications for LAA closure were a history of intracranial haemorrhage (32.4%), history of ischemic stroke during anticoagulant therapy (4.4%), high risk of bleeding (23.4%), end-stage chronic kidney disease (19.1%), and chronic liver disease and labile INR (10.3%). However, two or more indications frequently coexisted, thereby highlighting the extreme frailty of these patients (Figure 1).

LAA morphology, established with a TEE and angiography combination, was as follows: 14 subjects (20.6%) having cactus type, 20 (29.4%) windsock, 17 (25.0%) chicken wing, and 17 (25.0%) cauliflower.

Successful LAA closure was achieved in 67/68 patients.

### 3.2. Procedure and Periprocedural Events

Two patients experienced major periprocedural complications. The first one had a periprocedural embolic stroke which improved after mechanical thrombectomy. Notably, any thrombotic formation had been shown by intraprocedural TEE. No neurological deficit was reported at the follow-up neurologic visit. The other patient developed massive intracranial haemorrhage as a consequence of dual APT. This patient was affected by cerebral amyloid angiopathy and experienced the event the day after the procedure, approximately 24 hours after the first administration of the antiplatelet therapies.

### 3.3. Pharmacological Therapy and Follow-Up

At discharge, only 32 patients (47.1%) were on dual APT. About 50% of the sample was discharged without any antithrombotic therapy (7.3%) or with a single antiplatelet drug (42.7%), as described in Table 2. Two patients were discharged with an anticoagulant therapy: one for a concomitant procedure of AF ablation and one for a recently diagnosed deep vein thrombosis. Both patients were treated with DOACs for three months. At three months follow-up visit, 73.6% of the patients did not receive dual APT, of whom 14.7% had no thrombotic therapy and 58.9% were on single antiplatelet therapy. At 12 months of follow-up, only 8 patients were receiving dual APT; the main indication for dual APT persistence was generally represented by the presence of a coronary stent.

Six patients had incomplete follow-up: 5 had the LAA closure less than one year ago and 1 patient was lost. During the follow-up (mean time was 1.4 ± 0.9 years), three patients out of 62 had late adverse effects. Two of them had device-related thrombus without clinical consequences and one had extracranial bleeding. Two device-related thromboses were detected accidentally at TEE in patients implanted with WATCHMAN device. This finding was apparently not related to antithrombotic therapy. Indeed, one patient was receiving dual APT (ticagrelor twice a day and aspirin) and one was receiving a DOAC at the appropriate dose. The first one was treated adding enoxaparin to dual APT; the other was treated replacing DOAC with enoxaparin. In both cases thrombus was dissolved after another month of therapy with no clinical consequences. The patient who developed extracranial bleeding was affected by haemophilia and was discharged without any antithrombotic therapy. Three patients died during follow-up, but none of the deaths were related to the procedure or device. Indeed, two patients died of cancer and one of hepatic failure.

### 3.4. Prevention of Thromboembolic and Haemorrhagic Events

**Figure 2:**
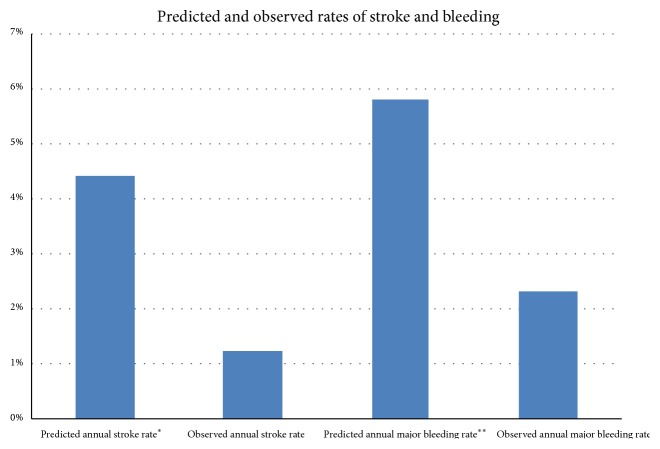
*Predicted and observed rates of stroke and major bleeding*. Effectiveness and safety of LAAO in reducing thromboembolic events and haemorrhagic complications. *∗*Calculated from CHAD_2_DS_2_-VASc score. *∗∗*Calculated from HAS-BLED score.

Considering the expected major bleeding rate of 6%, as calculated by HAS-BLED score, and the observed annual major bleeding rate of 2%, we obtained a 60% reduction in bleeding events (Figure 2). The expected annual risk of thromboembolism of the sample, according to the CHA_2_DS_2_-VASc score, was 4.4%. We recorded only one thromboembolic cerebral event in periprocedural setting and any cerebral event during the follow-up.

## 4. Discussion

We described a single-center cohort of patients who underwent percutaneous LAA occlusion.

Safety of the procedure was confirmed by the low frequency of early complications. Indeed, we achieved a successful device implantation in 67 out of 68 patients (98.5%), which is higher in comparison to the 91% in the PROTECT AF study and to the 95% in the CAP Registry [30], but comparable to the 98.5% of EWOLUTION registry [31].

Percutaneous LAA closure is a relatively safe technique with complications mostly related to the operator's experience [32]. In our Center, the procedures were performed only by two skilled operators, thus explaining the high frequency of successful device implantation.

The current instructions for use, provided by manufacturers and updated after publication of follow-up results of EWOLUTION trial [28], suggest at least three months dual APT after device implantation. The goal of antiplatelet treatment following implantation of intracardiac device is to prevent thrombus formation on its surface before its complete endothelialization [23, 25]. In our study, 2 patients had device-related thrombus (3.2%). This result is similar to those obtained in other studies and confirmed in a recent large meta-analysis, which showed an overall incidence of 3.9% with a low rate of neurological complications [33]. As reported by other trials and registries [28, 33, 34], the device-related thrombosis in our study was apparently not related to the antithrombotic therapy. Indeed, one patient was receiving dual APT for acute coronary syndrome and the other one was receiving OAT for deep venous thrombosis.

About 50% of the population study was discharged without any antithrombotic therapy or with a single APT and this proportion grew to 73.6% at three-month follow-up visit. Nevertheless, our rate of device-related thrombosis is similar to those obtained in other studies with higher prescription rates of dual APT. For example, in EWOLUTION trial [28] the average time to dual APT discontinuation was 6 months and only 13% of patients were discharged on single antiplatelet therapy or with any antithrombotic drug. Similar observations have been made in studies on antithrombotic therapy after transcatheter aortic valve implantation and a recent large meta-analysis confirmed the lack of benefit from dual APT compared to single APT in these patients [35]. This and other recent observations [36] suggest that dual APT following devices implantation may be an overtreatment. A possible explanation is that the mechanism leading to device thrombosis in the left atrium is different from those that cause stent thrombosis in coronary arteries. Indeed, while coronary stent thrombosis is linked to a high shear stress with consequent platelet activation, the left atrium thrombosis is associated with a very low shear stress with the formation of a fibrin-rich clot [37]. Thus, it is possible that dual APT, that is extremely effective in preventing coronary stent thrombosis, could have only a small impact in preventing atrial device thrombus formation, causing rather an increase in bleeding.

This exploratory hypothesis requires large confirmatory trials that compare dual APT, single APT, and OAT following the LAA occlusion.

The very low rate of antithrombotic drugs prescription in our study was due to patient selection. Indeed, population study was at very high bleeding risk. Mean HAS-BLED score was 3.2 ± 1.0 and the proportion of patients with a HAS-BLED score ≥3 was 73.5% (comparing the EWOLUTION trial [31], mean HAS-BLED score was 2.3 ± 1.2 and proportion of patients with a HAS-BLED score ≥3 was 40%). Another relevant result of our study is the 60% reduction in the observed bleeding rate. Confirming previous data, our findings showed that LAA occlusion is associated with a significant reduction in bleeding [29, 36], mainly driven by the discontinuation of antithrombotic drug.

### 4.1. Limitations

We acknowledged some limitations of this study. First, the lack of randomization does not allow a control group treatment and the comparison of events (bleeding and ischemic stroke) has been based on estimated scores. In addition, the relative short period of follow-up might increase the possibility of underestimating event rates, although it is plausible that the first month after the procedure represents the most critical period after LAA closure.

## 5. Conclusions

LAA occlusion is feasible and safe and prevents stroke in patients with AF with contraindication to OAT. Although it is possible that after LAA occlusion single antiplatelet therapy is effective as dual antiplatelet therapy, specific studies on the topic are needed.

## Figures and Tables

**Table 1:** CHAD_2_DS_2_-VASc and HAS-BLED scores.

Value	CHAD_2_DS_2_-VASc	HAS-BLED
0	1 (1.5)	N/A
1	N/A	1 (1.5%)
2	15 (22.1%)	17 (25.0%)
3	14 (20.6%)	26 (38.2%)
4	17 (25.0%)	15 (22.1%)
5	16 (23.5%)	9 (13.2%)
6	3 (4.4%)	N/A
7	2 (2.9%)	N/A

Mean	3.7 ± 1.4	3.2 ± 1.0
Predicted annual risk	4.4%	5.8%

**Table 2:** Antithrombotic therapy before and after left atrial appendage closure.

	Pretreatment	Posttreatment, discharge	Posttreatment, 3 months	Posttreatment, 6/12 months
n	68	68	68	62
No antithrombotic therapy	16 (23.5%)	5 (7.3%)	10 (14.7%)	20 (32.3%)
Single anti-platelet therapy	12 (17.7%)	29 (42.7%)	40 (58.9%)	34 (54.8%)
Dual anti-platelet therapy	23 (33.8%)	32 (47.1%)	16 (23.5%)	8 (12.9%)
Anticoagulant therapy	17 (25.0%)	2 (2.9%)	2 (2.9%)	0 (0.0%)

## Data Availability

The data used to support the findings of this study are available from the corresponding author upon request.

## Abbreviations

LAA: Left atrial appendage
LAAO: Left atrial appendage occlusion
AF: Atrial fibrillation
OAT: Oral anticoagulant therapy
VKA: Vitamin K antagonists
NOACs: Nonvitamin k antagonist Oral Anticoagulants
ACP: AMPLATZER™ Cardiac Plug
TEE: Transesophageal echocardiography.
